# Shifting Instead of Drifting – Improving Attentional Performance by Means of the Attention Training Technique

**DOI:** 10.3389/fpsyg.2019.00023

**Published:** 2019-01-23

**Authors:** Vincent Barth, Ivo Heitland, Tillmann H. C. Kruger, Kai G. Kahl, Christopher Sinke, Lotta Winter

**Affiliations:** ^1^Department of Psychiatry, Social Psychiatry and Psychotherapy, Hannover Medical School, Hanover, Germany; ^2^Division of Clinical Psychology and Sexual Medicine, Hannover Medical School, Hanover, Germany

**Keywords:** metacognitive therapy, attentional training technique, attentional performance, MCT, ATT, metacognition, healthy participants

## Abstract

**Background:** The Attention Training Technique (ATT) as part of Metacognitive Therapy (MCT) has shown to be a promising treatment element for several psychiatric disorders such as depression and anxiety. ATT predicts improvements of the ability to shift attention away from internal and non-relevant stimuli (e.g., ruminative thoughts) toward the relevant stimuli and aims to increase attentional flexibility and control. The current study investigated the impact of the Attention Training Technique on attentional performance.

**Methods:** Eighty-five healthy participants (29 in two doses ATT, 28 in four doses ATT and 28 in the control group; 18–37 years of age) were administered a test battery for attentional performance before and after an intervention of two doses ATT (23 min duration) vs. four doses of ATT (46 min duration) vs. a control condition (non-intervention audio file via headphones. The test battery measured selective attention, inhibition, working memory, and attentional disengagement and comprised the following tasks: dichotic listening, attentional bias, attentional network, stroop, 2-back and a 3-back.

**Results:** After ATT (both two and four doses), reaction time during dichotic listening was significantly faster compared to the control condition. Furthermore, reaction time to neutral stimuli in the attentional bias task was faster after four-doses ATT compared to two doses ATT and the control condition. We found a trend toward a reduced stroop effect for both ATT conditions compared to control group. There were no effects of ATT with regard to the attentional network task, the 2-back or the 3-back task.

**Conclusion:** This first empirical evidence suggests that ATT promotes specific attentional flexibility in healthy participants. Based on the same mechanism, ATT may have beneficial effects on attentional performance in clinical populations and might be a promising tool in both healthy and clinical participants.

## Introduction

Attention is a central function of neural processing. In 1890, James stated: “Everyone knows what attention is. It is taking possession by the mind, in clear and vivid form, of one out of what seem several simultaneously possible objects or trains of thought. Focalization, concentration of consciousness are of its essence. It implies withdrawal from some things in order to deal effectively with others” (James, 1890, pp. 403–404). Thus, attention enables the organism to prioritize some processes while ignoring less important ones. The Self-Regulatory Executive Function model (S-REF; Wells and Matthews, 1994) proposes that psychological disorders, e.g., depression or anxiety, develop when the person’s style of thinking and coping leads to prolonged maladaptive emotional responses. These thinking and coping styles, e.g., rumination, worrying, threat monitoring etc., form the cognitive attentional syndrome (CAS; Wells, 2005; Fisher and Wells, 2009). Rumination as an active coping style leads to performance deficits and reduced flexibility of the cognitive system (Wells and Matthews, 1996). In addition, it is characterized by inflexible attention and the reduced ability to shift attention toward relevant stimuli (Whitmer and Banich, 2007).

Metacognitive Therapy (MCT) is a psychotherapeutic treatment that is based on the S-REF model of psychological disorder (Wells and Matthews, 1994, 1996). It aims to decrease the strength or remove the CAS entirely by changing metacognitive beliefs and re-establishing attentional flexibility (Wells, 2009).

The Attention Training Technique (ATT) is a component of the MCT manual with the aim to reduce the CAS by re-orienting attention, i.e., shifting away from self-focus (Wells, 1990, 2007). The ATTs main focus is to improve attentional control and attentional flexibility by combining three auditory attentional exercises: selective attention, attentional switching and divided attention (Wells, 2007). The aim of the ATT is to strengthen the ability to focus on demand and improve the ability to focus on multiple stimuli at the same time. ATT leads to increased attentional control and reduced ruminative thoughts (Papageorgiou and Wells, 2000). A recent meta-analysis has demonstrated the efficacy of ATT as a stand-alone treatment for depression and anxiety, yielding greater treatment gains than comparison groups (autogenic training, progressive muscle relaxation, etc, Knowles et al., 2016). There is initial evidence showing that the ATT improves self-regulation in children (Murray et al., 2016). This gives rise to the question of whether ATT has the ability to not only relieve symptoms, but also to improve attentional performance by increasing control and flexibility of attention. The aim of the present study is to determine whether cognitive performance and selective attention increases after ATT training in healthy participants. Whereas therapeutic effects of ATT have been demonstrated by several studies, there is little knowledge about which specific neuropsychological domains are affected by the training. Therefore, the present study investigates which domains of attentional performance may be improved after ATT in healthy participants and the amount of training needed to gain effects.

## Materials and Methods

The sample consisted of 85 healthy students recruited from a German university. Participants confirmed to be free of psychiatric diagnoses according to ICD-10 Criteria in the last 3 month. Data of four participants were discarded due to incomplete or invalid recordings, resulted in a total sample size of *n* = 81. The sample was between 18 and 37 years of age (mean age: 23.7, *SD* = 3.6). 64.2% of participants were female, 35.8% were males. All study procedures were approved by the local medical ethical committee. All participants provided written informed consent in accordance with the Declaration of Helsinki. Participants received monetary compensation for participation.

### Procedure

**FIGURE 1:**
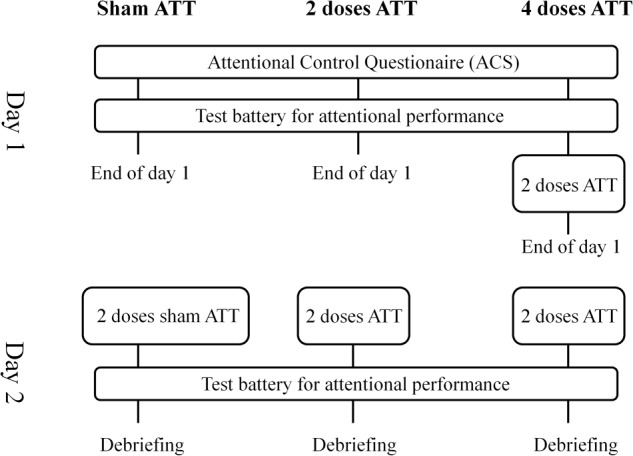
Procedural overview of current study design. Displayed are the three groups (sham ATT, 2 doses ATT and 4 doses ATT) and their procedure during the two consecutive days of the experiment. The test battery for attentional performance consists of dichotic listening, attentional network task, emotional dot probe, stroop task, 2-back and 3-back.

Participants were randomly assigned to one of three groups: two doses ATT (*n* = 27), four doses of ATT (*n* = 27) and the active control group sham training (*n* = 27, for a detailed description see *sham training*). The procedure took place on two consecutive days (see Figure 1 for the procedural overview). The participants sat in front of a 19 inch LCD-Screen (Samsung Syncmaster 914n) with Sennheiser HD 558 over-ear headphones. Participants first received general information regarding the experiment and subsequently provided written informed consent. Participants then completed a German version of the attentional control questionnaire (ACS, Derryberry and Reed, 2002). Afterward, all groups performed a test battery to assess attentional performance. This completed the experimental procedures for the two doses ATT and the active control group on day 1. The group of four doses ATT completed two training session of ATT using an audio file (23 min., for a detailed description of the audio file see *ATT*) after the test battery. Experiment length on the first day was approximately 42 min for two doses ATT and the control group and 65 min for the 4 doses ATT condition. On day 2, the two doses and four doses ATT groups started with listening to two training sessions of ATT (23 min). The control group listened to the control treatment (22 min, see *sham ATT*). Then, every group performed the attentional performance test battery as described below directly after the training session. The experiment ended with a debriefing. The total experiment length on day 2 was approximately 55 min for all groups. All cognitive tasks and the delivering of the audio files were programmed using neurobehavioral systems presentation^®^ software version 18.3 (Neurobehavioral Systems, Inc., Berkley, CA, United States).

### Measures

The attentional performance test battery comprised six well-validated tasks. Tasks were presented in the order of the following description with short breaks in between. Every test started with a short exercise block to ensure participants followed the instructions of the tasks. After the training run at the start of each task, the instructions were repeated to ensure that participants understand the task correctly.

### Dichotic Listening

The first task was the dichotic listening task as described in Asbjørnsen and Hugdahl (1995). We used the dichotic listening task to measure whether ATT improved selective attentional focusing in the domain of auditory processing. Six consonant-vowel syllables (ba, da, ga, ka, pa, and ta) were presented at the same time via audio files over headphones on both ears (each ear one syllable), with a total of 36 different combinations. Participants were instructed to focus on the relevant target on the preferred sides or forced listening condition (only the left or only the right side) and ignore the other stimuli. Syllables were presented in identical (e.g., left: ba / right: ba) or different combinations (e.g., left: ta / right: da). Instructions were given to determine the syllables in three conditions: freely identifying only one of both presented stimuli (free choice), identifying stimuli on the left ear (only left) and identifying stimuli on the right ear (only right). Participants were required to indicate the correct stimuli by pressing the first letter of the relevant syllable (B, D, G, K, P, or T) on the computer keyboard as quickly as possible. 36 trials (every possible combination of the syllables) were presented in each condition, with randomized variable intertrial interval (varied systematically from 750 to 1125 ms, *M* = 1000 ms).

Due to incomplete or invalid recordings, group sizes in the analyses were: sham ATT (*n* = 26), two doses ATT (*n* = 27), four doses ATT (*n* = 27). The Outcome variable was the weighted mean of all left and right ear correct reaction times in milliseconds in the forced listening condition. The T2-T1 difference of these weighted means were subject to analyses.

### Attentional Network Task

The attentional network task was used to assess performance within three domains of attention: alerting, orienting an executive control (Fan et al., 2005). Consistent with Fan et al. (2005), we used three cue conditions (no cue, center cue, spatial cue) and two target conditions (congruent and incongruent). A fixation cross was displayed at the center of the screen during the whole trial against a gray background. The trial started with showing either no-cue (fixation cross remained unchanged) or middle cue (asterisk on the position of the fixation cross) or spatial-cue (asterisk above or below the fixation cross, on the position were the target appears) for 200 ms. Then the cues disappeared and a jittered pause (300–1050 ms, *M* = 495 ms) with only showing the fixation cross followed. The target stimuli consisted of a row of five black arrows pointing either left or right, displayed either below or above the fixation cross and participants were instructed to indicate the direction of the middle arrow. This arrow in the middle was flanked on both sides by two arrows in the same direction (congruent condition) or in the opposite direction (incongruent condition). Target and flanker arrows were presented for 1600 ms. Participants had to identify the direction of the centrally presented arrow by pressing the identical arrow buttons (left or right) on the computer keyboard. After giving a response, the arrows disappeared and only the fixation cross was presented for the intertrial interval for 1600 ms (range 800–2000 ms). Participants had to shift spatial attention from the fixation point to the target stimulus in each trial in order to determine the proper response (Fan et al., 2005). Font size of the arrows was 60 and 55 for the asterisks. The three cue condition allowed to measure alerting and/or orienting benefits by giving no cue (baseline), middle cue (alerting, temporally informative) and spatial-cue (alerting plus orienting, temporally, and spatially informative). 120 trials were presented.

Due to incomplete or invalid recordings, group sizes in the analyses were: sham ATT (*n* = 23), two doses ATT (*n* = 23), four doses ATT (*n* = 21). The five outcome variables were mean reaction times of correct hits (for the no cue, the middle cue and the spatial cue conditions), and mean reaction times for congruent and incongruent stimuli, which were conducted for alerting, orienting and executive control as described by Fan et al. (2005). The T2–T1 difference of these weighted means were subject to analyses.

### Emotional Dot Probe Task

The emotional dot probe task was used to measure selective attentional control in the visual domain. Similar to Donaldson et al. (2007), a permanent central fixation cross on the computer screen was presented with a word pair (one word above, the other below the central fixation point) displayed for 1000 ms. This was followed by a fixation cross for 400 ms. The target (asterisk) presentation appeared in the position of one of the words for 2 s. in each trial, one word had a negative valence and the other was neutral. Words were taken from the ANGST-Database (Schmidtke et al., 2014). Neutral words had a valence between -0.2 and 0.2, emotional words had a valence of <-2. Participants were required to indicate the position of the asterisk as quickly as possible by pressing one of two response buttons (left for the word above and right for the word below) on the computer mouse. Font size of the words was 65 and 55 for the asterisks. The target was either presented on the position of the emotional word or the neutral word. Fifty trials were presented per condition. The intertrial interval was jittered around 750 ms (range 500–1000 ms). Two conditions were recorded: asterisk in the position of the neutral or emotional word, whereas the neutral word condition stands for the attentional disengagement from the emotional word toward the asterisk in the position of the neutral word.

Due to incomplete or invalid recordings, group sizes in the analyses were: sham ATT (*n* = 23), two doses ATT (*n* = 24), four doses ATT (*n* = 23). Outcome variables were the mean reaction times for neutral and the emotional correct responses in milliseconds. Analyses in the emotional dot probe task were conducted by subtracting emotional reaction times minus neutral reaction times in order to reveal the costs of attentional disengagement. Analyzed were the T2-T1 difference of emotional minus neutral reaction times and neutral and emotional reaction times additionally.

### Stroop Task

The stroop task (Stroop, 1935) was used to measure selective attention and executive control as inhibition in the process of parallel distribution processing model (See MacLeod, 1991). Trials presented the capitalized color words RED, YELLOW, GREEN, and BLUE for 1 s against a black background. In congruent trials, words were presented in its matching hue [e.g., BLUE in blue, colors used in RGB space: red (255,0,0), yellow (255,255,0), green (0,255,0), blue (0,0,255)]. Incongruent trials showed color words in a mismatching hue of the other three colors (e.g., BLUE in green). Participants had to indicate the hue of the words and ignore the semantic meaning of the color words. Participants gave their answers by pressing four keys, colored in the four named colors, on the keyboard, on the position of the letters S (red), X (yellow), K (green) and M (blue). An intertrial interval which jittered around 1750 ms (range 1500–2000 ms) was set before the next trial started. Font size of the words was 80. A total of 100 trials were presented, equally distributed across conditions (i.e., 50 congruent and 50 incongruent trials).

Due to incomplete or invalid recordings, group sizes in the analyses were: sham ATT (*n* = 26), two doses ATT (*n* = 27), four doses ATT (*n* = 27). Outcome variables were the mean reaction times of congruent hits and mean reaction times of incongruent trials in milliseconds. To index inhibition, we subtracted incongruent stimuli reaction times from congruent stimuli reaction times in order to determine stroop effect costs. Additionally, T2-T1 difference of incongruent minus congruent reaction times and incongruent reaction times were analyzed.

### 2-Back / 3-Back

The N-back task was used to measure working memory (WM) performance as described in Braver et al. (1997). We used a sequential letter task in the version of 2-back and 3-back. Participants had to determine whether the current letter was identical to the previous letter two trials (2-back) or three trials (3-back) before (see Braver et al., 1997, p. 57 for detailed description). Each displayed letter was presented for 1500 ms, followed by a 500 ms pause before the next letter appeared. In each version participants had to identify a target letter and non-target letter by pressing two keys (X for targets, M for non-targets). All 26 alphabetical letters were used in a randomized order, with no more than two targets in a row. One 2-back exercise block containing 10 letters was presented before the 2-back and 3-back tasks started. In addition, an experimenter verbally instructed participants by giving examples for the 2- and 3-back tasks. This was done with the purpose of ensuring every participant had understood the task. A total of 150 letters in each n-back task was presented, with 50 targets and 100 non-targets.

Due to incomplete or invalid recordings, group sizes in the analyses were in 2-back: sham ATT (*n* = 25), two doses ATT (*n* = 25), four doses ATT (*n* = 27) and in 3-back: sham ATT (*n* = 26), two doses ATT (*n* = 26), four doses ATT (*n* = 26). Outcome variables were the means of hits of target and non-target reaction times in milliseconds. The T2-T1 difference of these weighted means were subject to analyses.

### ATT

The attention training technique was presented using a standardized audio file as described in the MCT manual (Callinan et al., 2014; Fergus et al., 2014). A German version of the ATT was used (available at http://www.metakognitivetherapie.de/). The audio file follows the instructions provided by the MCT manual (Wells, 2009). As described above, each training session consisted of hearing the ATT audio file twice. The first sound file included explanations often upcoming training (1 min), where the participants were instructed to focus a visual fixation point and not to suppress or avoid internal events (e.g., thoughts, emotions) while listening to the auditory stimuli. The ATT comprises three auditory attentional exercises and lasts 12 min in total. In the audio file six different sounds (a clock, church bells, bird song, insects, traffic and running water) are presented and a male voice gives instructions on what to focus the attention on. ATT audio file starts with selective attention (5 min), where the participants perceive instructions to give intense attention to a specific individual sound (e.g., the ticking of a clock) while resisting distraction by others. Participants are instructed to focus on the voice of the instructor and the six different sounds as well as sounds in the room around the person successively. The next part of ATT was the rapid attention switching (5 min), in which participants have to switch attention between different sounds and spatial locations with increasing speed as this phase progresses. The last exercise practices divided attention (1 min), in which participants have to expand the width and depth of their attention and attempt to process multiple sounds and locations simultaneously (Wells, 2009). After finishing the first ATT session, the subject had the option for a short break and afterward the task continued with another ATT session identical with the first session but without the initial explanation of the instructor (double training).

### Sham ATT

The control / sham training group listened to a non-treatment audio file (11 min each file, for two sessions 22 min total), which comprised the same six different sounds identical in order, duration and intensity as in the ATT, but without any verbal instructions (audio file is available at http://www.metakognitivetherapie.de/). As in the ATT conditions, participants had a short break after hearing the first round of the sham training.

### ACS

A German version of the Attentional Control Scale (ACS; Derryberry and Reed, 2002) is a self-report measure of attentional control and attentional shifting. It comprises 20 items rated on a 4-point Likert scale (almost never, sometimes, often, always). The questionnaire measures the general capacity for attentional control. High scores on the ACS represent a good capacity in effortful attentional control, with the subscales of focused attention, shifting attention and attention flexibility. The Outcome variable was the total sum score of the ACS. The ACS score was included in order to control for potential confounding effects from pre-test attentional control ability.

### Statistical Analyses

Analyses were conducted with SPSS Statistics version 23.0 (IBM Corp., Armonk, NY, United States) using repeated measure General Linear Model (GLM) and repeated Analysis of Variance (ANOVAs) for each task. For all analyses reported hereafter, a *p*-value of <0.05 was considered significant. To investigate whether ATT vs. sham training affect the respective attentional performance domains, we conducted repeated measures ANOVAs using ATT (2 dosed and 4 doses combined) vs. sham training as a factor and the respective outcome parameters per test (T1 vs. T2) as dependent variables. *Age, gender*, and *ACS* total score were used as covariates for all further tests to correct for potential confounding effects.

## Results

### Sample Characteristics

There was neither a difference in age (*p* = 0.86) nor in ACS total score (*p* = 0.50) between all ATT groups. In addition, sex was evenly distributed across all groups [*x*^2^(2) = 0.75, *p* = 0.68]. There were no differences between the ATT (two and four doses combined) and sham training groups in age: (*p* = 0.98), ACS total score (*p* = 0.25) and sex [*x*^2^(1) = 0.61, *p* = 0.41]. In addition, all following significant and non-significant effects remained the same by excluding the covariates (*age, gender*, and *ACS* total score) from analysis.

### Dichotic Listening

**FIGURE 2:**
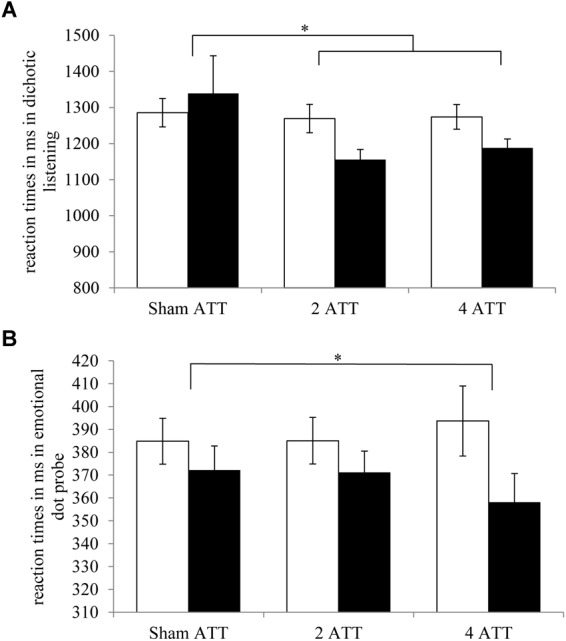
Reaction times for T1 (white) and T2 (black) per ATT group (sham ATT, two doses ATT, four doses ATT) are displayed for **(A)** Dichotic listening. **(B)** Emotional dot probe. Error bar indicate ±1 standard error of the mean (SEM). ^∗^*p* < 0.05.

The dichotic listening task was used to measure selective attentional focusing in the domain of auditory processing. Participants that received ATT were significantly faster in correctly responding than the sham training group (T2–T1) [*F*(1,75) = 5.17, *p* = 0.026, η_p_^2^ = 0.065; see Figure 2]. Further analyses showed no differences between four and two doses ATT in reaction times (*p* = 0.59).

### Emotional Dot Probe

The emotional dot probe task was used to measure selective attentional control in the visual domain. There was no difference between ATT and sham ATT (T2–T1) in emotional minus neutral reaction times (*p* = 0.89). The ATT group (two and four doses combined) did not differ from the sham training group in neutral reaction times (*p* = 0.19) or in reaction times of emotional reaction times (*p* = 0.19).

There was no significant difference between four doses of ATT when compared to the sham training group in emotional minus neutral reaction times (*p* = 0.59). However, participants in the four doses of ATT group responded significantly faster to neutral stimuli in comparison with the sham ATT group [*F*(1,42) = 4.97, *p* = 0.031, η_p_^2^ = 0.106, see Figure 2]. Furthermore, there was a trend toward faster reaction times for the four doses of ATT group vs. sham ATT with regard to emotional stimuli [*F*(1,42) = 3.22, *p* = 0.08, η_p_^2^ = 0.071]. Two and four doses of ATT did not differ significantly with regard to the neutral reaction times. However, there was a trend showing that the group that received four doses of ATT were slightly faster in reacting to corresponding stimuli than the two dose ATT group *F*(1,42) = 2.97, *p* = 0.092, η_p_^2^ = 0.066).

### Stroop

The stroop task was used to measure selective attention and executive control as inhibition. There was a trend for faster reaction times (incongruent – congruent reaction times) in participants that received ATT compared to the sham ATT group [*F*(1,75) = 3.12, *p* = 0.081, η_p_^2^ = 0.040]. Two and four doses of ATT did not differ from each other with regard to stroop task costs (*p* = 0.9). Analyzing the incongruent reaction times, ATT group and sham ATT group did not differ from each other (*p* = 0.246). Additional, there was no group difference in incongruent reaction times between two and four doses ATT (*p* = 0.534).

### ANT

The Attentional network task was analyzed to measure alerting, orienting and executive control. There was no difference between ATT groups and sham training with regard to alertness (*p* = 0.28), in orienting (*p* = 0.53) or in executive control (*p* = 0.92).

### 2-Back / 3-Back

The N-back task was used to measure working memory performance. 2-back and 3-back were analyzed by the means of hits of targets reaction times in milliseconds. ATT group and sham training showed no difference in reaction times of hits (*p* = 0.77) nor number of correct hits for the 2-back task. There was no difference between ATT group and sham ATT in non-targets reaction times in the 2-back task (*p* = 0.3). Furthermore, there was no differences with regard to 3-back reaction times of hits between ATT group and sham ATT (*p* = 0.55) nor number of hits. There was no difference between ATT group and sham ATT in non-targets reaction times in the 3-back task (*p* = 0.48).

## Discussion

This study examined the impact of the ATT, a standalone treatment of the MCT manual, on attentional performance in a randomized single blinded pre-post comparison of two doses ATT, four doses ATT and sham ATT. We showed that participants who received ATT were faster in auditory selective attention during dichotic listening. In addition, participants who received four doses of ATT performed faster in attentional disengagement in the emotional dot probe task compared to sham training. Furthermore, there was a trend toward faster reaction times in participants who received ATT compared to sham ATT in responding to stroop effect costs. There were no effects of ATT with regard to the ANT and the 2-back/3-back tasks.

The attention training technique showed a positive impact on auditory selective attention. Two and four doses of ATT yielded faster responses when identifying syllables on the right or left side while ignoring the irrelevant stimuli on the contralateral ear. This demonstrates that ATT induces near transfer effects in the trained domain (i.e., auditory attention). Our findings suggest that attentional flexibility through improving selective attentional control can be trained via only two doses of ATT. There was a significant improvement regarding attentional disengagement from emotional stimuli of participants with four doses ATT as measured with an emotional dot probe task. This provides evidence for a specific training effect of disengagement in the domain of the visual attention and a transfer effect from the auditory to the visual modality. Training with ATT leads to increased attentional flexibility in the form of faster disengagement of attention from irrelevant and/or emotional stimuli toward relevant stimuli. Consistent with initial evidence for attentional disengagement from negative stimuli (see Callinan et al., 2014), we found additional support for a growth of attentional flexibility. These findings are consistent with the theory of MCT and ATT, hypothesizing an improvement of attentional control (Wells, 2009).

As a further outlook, ATT may not only reduce clinical symptoms as shown by Knowles et al. (2016), but also improve attentional performance in healthy and clinical population. In fields aiming to improve attentional abilities (e.g., high-performance athletes) or reduce deficits (disorders related to the CAS) it may be useful to train attentional control via ATT. For example, as stated by Wells (2007) ATT could reduce auditory hallucinations, as evident from two case studies (Valmaggia et al., 2007; Levaux et al., 2011). Training attentional flexibility could allow less maintenance of auditory hallucinations or ruminative thoughts. The current study provides a first step toward understanding the mechanisms of ATT and its effect on healthy populations.

In the domain of selective attention and executive control, operationalized by the stroop task, only a trend of ATT was shown. Possibly for ATT, more training sessions or more statistical power would be necessary to determine an effect in the domain of subject’s ability to deflect task irrelevant information. This could be in line with the identified dose effect in the emotional dot probe task, and its similar underlying mechanism of blending out irrelevant task information. As the stroop measures not only selective attention but also executive control (see MacLeod, 1991), ATT training of two or four doses could enhance selective attention but not executive control in the sufficient amount to determine group differences in the stroop task data. Taken together, training ATT may not improve cognitive abilities in general, but rather the specific capacity of attentional disengagement.

We found no evidence for an ATT effect in the domain of the attentional network based on alerting, orienting and executive control. The theoretical background of alerting is defined by Posner and Petersen (1990) as achieving and maintaining an alert state. This domain is not defined as the main aim of ATT and therefore possibly explains the lack of training impact. Whereas orienting (selection of information from sensory input) and executive control (resolving conflict among responses) could be reasonably defined as a potential aim of ATT, both networks might be too close to the level of automatic processing and therefore not be modified through ATT as based on the S-REF-model (see Wells and Matthews, 1994). Furthermore, in comparison with mean reaction times of the Fan et al. (2005) sample (*n* = 16) of adults ranging from 18 to 36 years (e.g., congruent reaction times hits: *M* = 717 ms, *SD* = 110), our sample has demonstrated faster pre-training reaction times (congruent reaction times hits: *M* = 440 ms, *SD* = 44 ms). Hence, this study sample might be different as it consists of a better trained student sample. Furthermore, the Fan et al. (2005) study recorded the ANT using 228 trials to determine alerting, orienting and executive control, whereas our study used 120 trials and approximately 10 min of duration. Implementation might be too brief to record potential training effects in the three domains of attention. Further, four doses of ATT might be too little training to detect potential effects of ATT on the three parameters of the ANT.

We found no evidence for an effect of ATT in the domain of WM measured by the 2-back and 3-back tasks. 2-back and 3-back tasks were included in this study in order to determine whether ATT related training effects might be observed in the area of WM. Research regarding the domains of WM has shown that it consists, besides attentional control, of primary and secondary memory (Shipstead et al., 2014). Consistent with the idea that ATT improves attentional control rather than WM, our data suggests that WM performance does not seem to be trained significantly through ATT.

Here, we successfully demonstrated the training effect of ATT on healthy participants. However, the statistical power of the current study is limited by its sample size (*n* = 81). Future studies should incorporate larger samples to enhance statistical power. In addition, increasing the number of trials could result in more accurate estimation of the training effect. We omitted using longer tasks as to not overstrain the cognitive resources of the participants as the overall cognitive load was already quite high (23 min training/sham training + six different tasks). Due to multiple comparisons in statistical analysis there could be increased Type I errors. As this study was exploratory, providing an overview of those domains potentially affected by ATT, confirmatory studies are necessary to validate the present results.

We found preliminary evidence for a dose-dependent effect of ATT. Data suggests that four doses of ATT yielded greater training responses than two doses. Whereas previous clinical studies investigated doses ranging from 1 to 11, Knowles et al. (2016) conclude that one to two doses ATT could yield immediately measurable effects in symptom reduction. Symptoms reduced substantially after three doses of ATT and remained stable throughout the trial of additional ATT doses. With regard to the effect of ATT on attentional performance, we found evidence for an effect of only two doses of ATT. While more studies with a wider range of ATT doses are necessary to determine the optimal dose of ATT, the current findings suggest that more than two doses ATT should be applied. Our study investigated the direct effect of ATT with the performance test directly following the last training session. While a direct effect of ATT was demonstrated, we cannot assess at present whether there is only a temporary effect of ATT on attentional performance. Clinical case-studies using a follow-up design with a dose range of 6 to 11 ATT sessions suggest sustained effects of ATT on symptoms after 6 or 12 months, respectively (Knowles et al., 2016). To our knowledge, no follow-up studies have been conducted regarding ATT induced improvements in the domain of auditory and visual attentional control. Future studies should also investigate the duration of the observed effects and the ideal dose of ATT.

It might be worthwhile to investigate the corresponding clinical and neurological correlates of the demonstrated ATT-based attentional performance effects. It would be interesting to evaluate if training effects are larger in a clinical population than in healthy participants. Such studies would allow to evaluate if attentional control and flexibility mediate the reduction of symptoms and where it is related to the mechanism behind observed reduced clinical symptoms after training ATT. Taken together, this study is the first to show that ATT has a positive impact on attentional performance using an elaborate sham control condition. This suggests ATT as a promising tool to improve attentional performance.

## Ethics Statement

This study was carried out in accordance with recommendations of the ethics committee of the Hannover Medical School with written informed consent from all subjects. All subjects gave written informed consent in accordance with the Declaration of Helsinki. The protocol was approved by the ethics committee of the Hannover Medical School.

## Author Contributions

All authors designed the original concept. VB recruited and instructed the participants under IH supervision. CS designed the experiments. VB, IH, and CS were responsible for data collection and data analysis. All authors wrote the manuscript.

## Conflict of Interest Statement

The authors declare that the research was conducted in the absence of any commercial or financial relationships that could be construed as a potential conflict of interest.
